# Glycated Hemoglobin and Incident Type 2 Diabetes in Singaporean Chinese Adults: The Singapore Chinese Health Study

**DOI:** 10.1371/journal.pone.0119884

**Published:** 2015-03-16

**Authors:** Michael P. Bancks, Andrew O. Odegaard, Woon-Puay Koh, Jian-Min Yuan, Myron D. Gross, Mark A. Pereira

**Affiliations:** 1 Division of Epidemiology and Community Health, School of Public Health, University of Minnesota, Minneapolis, MN, United States of America; 2 Duke-NUS Graduate Medical School Singapore, Singapore, Singapore; 3 Saw Swee Hock School of Public Health, National University of Singapore, Singapore, Singapore; 4 University of Pittsburgh Cancer Institute, Division of Cancer Control and Population Sciences, Pittsburgh, PA, United States of America; 5 University of Pittsburgh, Department of Epidemiology, University of Pittsburgh, Graduate School of Public Health, Pittsburgh, PA, United States of America; Medical College of Soochow University, CHINA

## Abstract

**Background:**

The American Diabetes Association recently included glycated hemoglobin in the diagnostic criteria for diabetes, but research on the utility of this biomarker in Southeast Asians is scant. The aim of this study was to evaluate the association between percent HbA1c and incident diabetes in an Asian population of adult men and women without reported diabetes.

**Methods:**

Data analysis of 5,770 men and women enrolled in the Singapore Chinese Health Study who provided a blood sample at the follow-up I visit (1999–2004) and had no cancer and no reported history of diabetes or cardiovascular disease events. Diabetes was defined as self-report of physician diagnosis, identified at the follow-up II visit (2006–2010).

**Results:**

Hazard ratios (and 95% confidence intervals) for incident diabetes by 5 categories of HbA1c were estimated with Cox regression models and continuous HbA1c with cubic spline analysis. Compared to individuals with an HbA1c ≤ 5.7% (≤39 mmol/mol), individuals with HbA1c 5.8–5.9% (40–41 mmol/mol), 6.0–6.1% (42–43 mmol/mol), 6.2–6.4% (44–47 mmol/mol), and ≥ 6.5% (≥48 mmol/mol) had significantly increased risk for incident diabetes during follow-up. In cubic spline analysis, levels below 5.7% HbA1c were not significantly associated with incident diabetes.

**Conclusions:**

Our study found a strong and graded association with HbA1c 5.8% and above with incident diabetes in Chinese men and women.

## Introduction

After critical review by an expert committee, the American Diabetes Association recently recommended the inclusion of percent glycated hemoglobin (HbA1c %) in the diagnostic criteria for diabetes [1,2]. HbA1c reflects average glucose concentrations over the previous 2–3 months and is predictive of microvascular complications in diabetes [1]. Advantages of HbA1c include it being a standardized test not requiring individuals to be of fasting state, nor does it impose lengthy time commitment or great discomfort [3]. This may lend the test to be used in lieu of other glycemic testing methods, particularly in settings not amenable to individual behavior or temperament.

Evaluation of HbA1c across populations is necessary to further understanding, especially with the global increases in diabetes and pre-diabetes [4]. Level of HbA1c is shown to predict incident diabetes in a variety of populations, but there is limited data from Southeast Asian populations [5]. Total diabetes cases and disease rates have reached epidemic proportions in Asia.^4^ Compared to persons of European decent, individuals from Asia tend to develop diabetes at a younger age and lower level of body mass index (BMI) [6]. This indicates there is a continuum of risk for developing the disease at levels not typically thought detrimental to Western populations. Across ethnicities in Asia, differences in average HbA1c level have been identified [7]. Thus, it is important to characterize the relationship between HbA1c and incident diabetes in Asian populations, particularly at levels of HbA1c considered “prediabetic” by Western standards.

Demarcation of increased risk for diabetes according to HbA1c level has clinical importance, especially in Southeast Asian adults where there is a dearth of literature on the topic, yet the population is high risk. The aim of this study was to examine the association of HbA1c and incident diabetes in this Chinese population in Southeast Asia by a priori interval of HbA1c and also by continuous HbA1c value to better illustrate the HbA1c-diabetes risk dynamic in this population.

## Methods

The Singapore Chinese Health Study (SCHS) cohort was drawn from men and women, aged 45 to 74 years, who identified with one of the major dialect groups (Hokkien or Cantonese) of Chinese in Singapore. From April 1993 through December 1998, 63,257 individuals were enrolled, approximately 85% of the eligible subjects who were invited to participate, and followed prospectively. Participants were residents of government-built housing estates, where 86% of the Singaporean population resided during the enrollment period. Subjects provided written informed consent with the completion of an in-person interview conducted in the participant’s home that included questions on demographics, height and weight, use of tobacco, usual physical activity, menstrual and reproductive history (women only), medical history, and a 165-item food frequency questionnaire assessing usual dietary intake for the previous year. Approval of the study protocol and procedure of consent were granted by the Institutional Review Boards at the National University of Singapore, University of Pittsburgh and the University of Minnesota.

### Collection of participant characteristics

Subjects were followed-up in 1999–2004 with a telephone interview (F1), during which subjects were asked to update their baseline interview information. Individuals were queried regarding smoking status (commencement and cessation) and regular consumption of alcoholic beverages (beer, wine, western hard liquor and Chinese hard liquor). Participants were asked to choose from eight frequency categories and four portion sizes and levels of alcohol intake were expressed in units of ‘drinks’ per week to facilitate comparison with western populations. One drink was defined as 375 ml of beer (13.6 g of ethanol), 118 ml of wine (11.7 g of ethanol), and 30 ml of western or Chinese hard liquor (10.9 g of ethanol). Other risk factors assessed at F1 include educational attainment (highest level completed), self-reported hypertensive status (yes/no) as diagnosis by physician, and body mass index (BMI) calculated with self-reported height (m) and weight (kg) as kg/meter-squared. For this analysis, participant characteristics reflect those given at the F1 interview.

### HbA1c analysis

Red-blood cells (RBCs) were isolated from whole blood and frozen until analysis that was performed at University of Minnesota. Percentage of HbA1c was analyzed in a Clinical Laboratory Improvement Amendments-certified laboratory using an automated high-performance liquid chromatography method where whole blood samples are treated with ethylenediaminetetraacetic acid on a Tosoh G7 hPLC Glycohemoglobin Analyzer (Tosoh Medics, Inc., San Francisco, California). Using the standards developed in the National Glycohemoglobin Standardization Program, this method for assessing the percentage of HbA1c was calibrated to the reference range of HbA1c 4.3%-6.0% with a coefficient of variation range 1.4%-1.9% [8].

### Diabetes assessment

Individuals were evaluated for diabetes status at baseline, F1, and F2. Self-reported diabetes as diagnosed by physician was assessed with the following question: “Have you been told by a doctor that you have diabetes (high blood sugar)?” If yes: “Please also tell me the age at which you were first diagnosed?” Participants with a history of diagnosed diabetes at baseline or F1 were excluded from analysis. Individuals were considered to have incident diabetes if they reported developing diabetes anytime between the F1 interview and F2 interview. A validation study of the incident diabetes mellitus cases in the original cohort used 2 different methods, previously reported by Odegaard et al [9,10].

### Formation of analysis cohort

**Fig 1:**
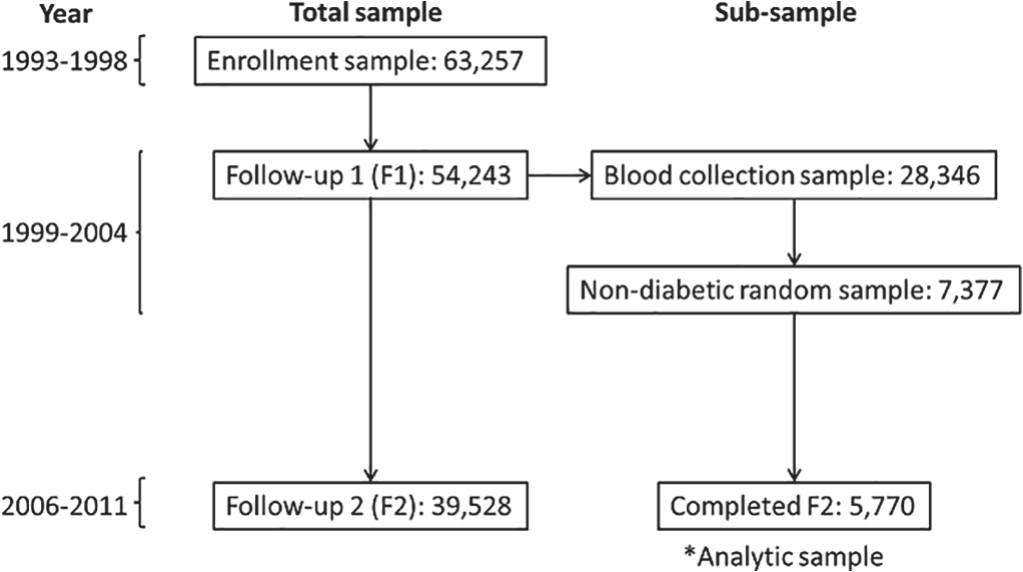
Timeline and assembly of sub-sample, The Singapore Chinese Health Study.

The formation of the current analytic sample is summarized in Fig. 1. Of the 54, 243 participants who were alive and participated at F1, a total of 28, 346 individuals provided consent to collect blood samples (roughly a consent rate of 65%). Participants in this analysis were individuals with no history of cancer at baseline or F1, and did not report a history of diabetes or cardiovascular disease at either interview. They were randomly selected and frequency matched to incident cases of type 2 diabetes from the full study population occurring between baseline and the follow-up interview on age (±2), time of blood draw, gender, and dialect (N = 7,377). This sub-sample was established in order to serve as a non-diabetic comparison group in future SCHS Genome Wide Association analyses of incident diabetes cases occurring prior to F1. This analytic sub-sample is slightly younger, more male, and reported more smoking, but is similar in respect to dialect, alcohol consumption, education, and BMI to their contemporaries participating at F1 who did not report diabetes, cardiovascular disease, or cancer. Linkage with the nation-wide registry of birth and death in Singapore reported 620 individuals from this sample died before a second follow-up interview that occurred in 2006–2010 (F2). These individuals tended to be older, male, of lower educational status, hypertensive, current smokers, and heavier consumers of alcohol compared to our final analysis cohort. Additionally, approximately 987 individuals from this sample could not be located to participate in the F2 interview as of the time of this analysis. These individuals tended to be older, reported a positive history of hypertension at F1, and were of lower educational attainment compared to those followed through the F2 interview. Both the group of individuals who died before F2 and those lost to the F2 interview had a higher average percent HbA1c compared to those who were in the final analytic sample. All participants with blood samples drawn and analyzed for HbA1c who participated in the F2 interview were included in this analysis regardless of HbA1c status for a final analytic sample of N = 5,770.

### Statistical analysis

HbA1c was divided into five categories to align with the distribution of the data, clinical relevance, and cut-points used in the literature. The categories were ≤ 5.7%, 5.8–5.9%, 6.0–6.1%, 6.2–6.4%, and ≥ 6.5%. Smoking status was classified as “never smoked”, “former smoker”, and “current smoker”. Alcohol was modeled continuously, average number of drinks consumed per week. In this analysis, education was characterized into 3 groups: no formal education; primary schooling; and secondary school or beyond. Participant characteristics were calculated across categories. Means and standard deviations were derived for continuous variables and proportions were calculated for categorical variables. Diabetes hazard ratios were calculated by HbA1c level. Crude and adjusted hazard ratios and 95% confidence intervals (CIs) were estimated with Cox proportional-hazards models. Traditionally a lower HbA1c level is beneficial in relation to development of diabetes and while HbA1c of 5.7% represents the lower bound for prediabetes diagnosis by the American Diabetes Association (ADA) criterion the HbA1c category of ≤ 5.7% was chosen as the reference category to provide a stable comparison group (cases > 10). The remaining categories of elevated yet normal HbA1c allow for comparison with other ethnic populations on diabetes risk in individuals with prediabetic levels of glucose and so potential clinically relevant ranges below the threshold for diabetes could be examined, unique to this population. The main effect crude model included only the HbA1c level. Adjusted models were constructed in this manner: Model 1 was adjusted for age, gender, dialect, and interview year. Model 2 was adjusted further for education, smoking status, and alcohol consumption. Model 3 was adjusted for all previous covariates plus BMI and hypertension status. When the above models included additional covariates including a baseline dietary pattern score (vegetable-fruit-soy or dim-sum-meat) [11] and average moderate (e.g., brisk walking, bicycling on level ground) and strenuous (e.g., jogging, bicycling on hills, tennis) physical activity per week, the results were not meaningfully changed. Therefore, the results with these additional adjustment variables were not presented. A variable for the squared HbA1c percentage was created for the assessment of quadratic relationship between HbA1c and risk of diabetes incidence. Restricted cubic spline analysis was performed centering the spline at HbA1c of 5.7%. Contributed person-time to the study was calculated as the duration from age at blood draw to age of reported type 2 diabetes diagnosis or age at administration of F2 interview (i.e., censoring date). Age and sex standardized incidence rates were calculated for each HbA1c level by categorizing age into three groups, ≤59, 60–69, and ≥70 years old and calculating sex specific crude incidence rates for each age group (cases/person years) according to the person-time, gender, and age distribution of the SCHS. To investigate if HbA1c levels differed across levels of sex, smoking, BMI, or age, separate multiplicative interactions were tested by adding product terms to the proportional hazards model. The proportional hazards assumption was assessed by HbA1c category and no violations were detected. Sensitivity analysis was also performed, by excluding observation in the first 3 years post blood draw for all 5,770 individuals, for purposes of accounting for possible sub-clinical disease or underlying poor health. Model discrimination was evaluated using Harrell’s C statistic. Statistical analysis was performed using SAS statistical software version 9.3 (SAS Institute Inc., Cary, NC) and STATA 12.0 software (StataCorp LP., College Station, Texas).

## Results

Over 50% of the study population had HbA1c ≤ 5.7%. Among the remaining HbA1c categories the study population distribution in ascending order was 20%, 12%, 7%, and 5%, respectively. Characteristics of the 5,770 participants analyzed are presented in Table 1. At F1, the sample average age was 62.9 years with a mean BMI of 22.9 kg/meter-squared. HbA1c was positively associated with age, BMI, current smoking, and self-reported hypertension. Average weekly alcohol consumption was similar across HbA1c categories as was dialect group. Females were slightly more represented than males across HbA1c categories with the exception of HbA1c ≥ 6.5% where female gender represented less than half of the category. HbA1c was inversely associated with highest educational attainment.

**Table 1:** Participant characteristics diabetes according to category of glycated hemoglobin (HbA1c%), The Singapore Chinese Health Study.

		HbA1c (%) Category						Excluded	
		≤ 5.7	5.8–5.9	6.0–6.1	6.2–6.4	≥ 6.5	P for trend	Died before F2	Lost to follow-up
Number		3262	1127	667	397	317		620	987
HbA1c %		5.4 (0.27)	5.8 (0.05)	6.0 (0.05)	6.3 (0.08)	7.3 (1.23)	< 0.001	5.96 (0.85)	5.90 (0.85)
Number of cases		11	16	13	11	63	< 0.001	—	—
Age		62.5 (7.1)	63.1 (7.0)	63.5 (7.0)	64.5 (7.0)	64.4 (7.6)	< 0.001	68.8 (7.0)	64.0 (7.5)
Female (%)		53	52	54	56	44	0.22	40.2	55.7
Cantonese dialect (%)		49	50	49	54	48	0.57	41.0	50.8
Education (%)							< 0.001		
	< Primary School	20	23	24	23	20		29.4	22.5
	Primary schooling	45	43	44	48	53		51.6	52.3
	≥ Secondary school	35	34	32	29	27		19.0	25.2
Smoking status (%)							< 0.001		
	Never	70	66	64	64	62		46.1	67.5
	Former	16	17	16	15	17		23.1	17.5
	Current	14	17	20	21	21		30.8	15.0
Alcoholic drinks/week		1.0 (4.0)	0.9 (3.6)	0.8 (3.4)	1.0 (4.0)	1.1 (4.6)	0.6	1.7 (5.8)	0.8 (4.0)
Physical Activity^1^ (min/week)		71.7 (177.1)	61.4 (156.0)	75.8 (192.7)	65.8 (139.8)	67.7 (198.9)	0.6357	NA	NA
BMI^2^ (kg/m^2)		22.5 (3.3)	23.0 (3.3)	23.3 (3.6)	24.3 (3.5)	24.3 (3.4)	< 0.001	22.9 (3.9)	23.0 (3.5)
Hypertensive (%)		29	32	37	42	44	< 0.001	41.9	40.2

^1^Physical activity is the average min/week of combined moderate and strenuous activity

^2^Body mass index

NA: Not applicable to estimate due to missing data

Continuous variables are means (SD)

Categorical variables are % within column

P for trend is testing for a linear trend across HbA1c category

**Fig 2:**
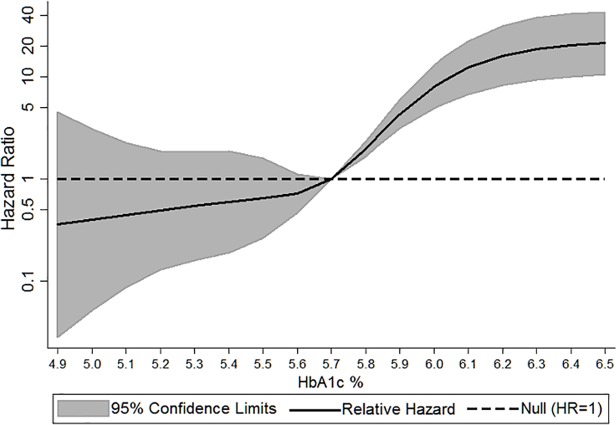
Cubic spline of the association between glycated hemoglobin (HbA1c %) and self-reported incident diabetes, adjusted for age, gender, and dialect, The Singapore Chinese Health Study. The hazard ratio (HR) is per each absolute increase of 1 percentage point in the glycated hemoglobin value at baseline. The shaded area is the 95% confidence interval from the restricted-cubic-spline model. The HR scale is logarithmic, the model is centered at the mean/median (5.7%), and the plot was truncated at the 5th and 95th percentiles of glycated hemoglobin (4.9% and 6.5%, respectively). The hazard ratio was adjusted for age, gender, and dialect.

The average follow-up time was 5.2 years. Individuals with HbA1c ≥ 6.5% had an average follow-up time of 4.7 years and 20% of this HbA1c category (n = 63) self-reported diagnosis of diabetes at F2. There were 114 incident cases of diabetes during the 29,753 years of total follow-up time, corresponding to a crude incidence rate of 383 cases per 100,000 person years. The overall age and sex standardized incidence rate was 415 cases per 100,000 person years. Presented in Table 2 are fully-adjusted hazard ratios (HR) with 95% confidence intervals and adjusted incidence rates according to HbA1c level. The HR for diabetes increased with higher HbA1c%. Compared to individuals with an HbA1c ≤ 5.7% (≤39 mmol/mol), individuals with HbA1c 5.8–5.9% (40–41 mmol/mol), 6.0–6.1% (42–43 mmol/mol), 6.2–6.4% (44–47 mmol/mol), and ≥ 6.5% (≥48 mmol/mol) had 4-, 5-, 8-, and 60-fold increase in risk for developing diabetes, respectively, after adjustment. The HRs for incident diabetes for each HbA1c category were attenuated but still strongly significant as the models were subsequently adjusted for potential confounders. The largest attenuation was observed for those with HbA1c ≥ 6.5%. The test for a quadratic association between HbA1c % and incident diabetes was statistically significant (p<0.001). Adjusted results from cubic spline regression are presented in Fig. 2. In spline analysis excluding individuals with HbA1c values in the lower and upper 5th percent (HbA1c < 4.9% and HbA1c ≥ 6.5%), risk of diabetes significantly increases for HbA1c > 5.7%, after adjusting for age, gender, and dialect group. Individuals with an HbA1c < 5.7% had reduced risk of incident diabetes compared to individuals with HbA1c of 5.7%, however the confidence limits span HR = 1. Formal tests of interaction were not significant for any characteristics (p > 0.10). Upon exclusion of observation of the first three years post blood draw the results did not substantively change, however the HRs in the two highest HbA1c categories did attenuate (shown in Table 2). These attenuated results should be interpreted with caution given the decrease in both sample size and amount of cases.

**Table 2:** Incidence rates and hazard ratios (HR) with 95% confidence intervals for incident type 2 diabetes according to category of glycated hemoglobin (HbA1c%), The Singapore Chinese Health Study.

	HbA1c (%) Category					
	≤ 5.7	5.8–5.9	6.0–6.1	6.2–6.4	≥ 6.5	
Number	3262	1127	667	397	317	
Incident cases (% total)	11 (10)	16 (14)	13 (11)	11 (10)	63 (55)	
Years follow-up (average)	16993 (5.2)	5793 (5.1)	3451 (5.2)	2018 (5.1)	1498 (4.7)	
Crude incidence rate	65	276	377	545	4206	
Standardized incidence rate	65	243	366	579	4935	Harrell’s C
Unadjusted HR model	1.0	4.30 (2.00, 9.27)	5.82 (2.61, 12.98)	8.54 (3.70, 19.71)	66.1 (34.8, 125.4)	0.860
Final adjusted HR model	1.0	4.17 (1.93, 9.00)	5.49 (2.45, 12.30)	8.25 (3.54, 19.25)	59.9 (31.1, 115.3)	0.876
Followed ≥ 3 years HR model	1.0	3.57 (1.56, 8.15)	5.11 (2.19, 11.90)	5.11 (1.92, 13.63)	38.3 (18.8, 78.0)	0.863

Crude incidence rate: per 100,000 person years

Standardized incidence rate: per 100,000 person years according to the gender, age and follow-up time distribution of the SCHS

Final adjusted model: Adjusted for age, gender, dialect, interview year, educational status, smoking status, average weekly alcohol intake BMI, and hypertensive status

Followed ≥ 3 years: analysis when excluding the first three years post blood-draw

## Discussion

In this study, Singaporean adult men and women with elevated, but not overtly diabetic levels of HbA1c were at increased risk for developing diabetes during follow-up. When looking at the spectrum of HbA1c values considered nondiabetic, there was strong, positive association with HbA1c values above 5.7%. These results are important, considering the difference in risk for developing diabetes by race and ethnicity [12]. Indeed, these results align with previous studies comprised of various race/ethnicities [5,13–15]. In distinct populations of US and European adult men and women, HbA1c strongly predicts incident diabetes [13,15]. In two older healthy populations of men and women, respectively, baseline HbA1c strongly predicted incident diabetes [16,17]. In a French population of adults with impaired fasting plasma glucose (FPG) at baseline (FPG ≥ 6.10 mmol/l), HbA1c predicted diabetes defined by future fasting plasma glucose (FPG ≥ 7.00 mmol/l) or diabetes medication use [18]. After controlling for baseline 2-hour glucose status, a 1% change in HbA1c was associated with nearly fourfold greater hazards of incident diabetes in a population of adult Hong Kong residents [19]. Notably, researchers did not adjust for potential confounders beyond baseline glucose (i.e., hazards unadjusted for age, sex, BMI, etc.) and participants in the study population of Hong Kong were, on average, 10 years younger than that of Singapore and naturally at lower risk for diabetes compared to the older SCHS sample. In a population of Japanese adult men and women, HbA1c independently predicted development of diabetes in 7 years of follow-up and an apparent multiplicative interaction between FPG and HbA1c on diabetes diagnosis was observed. This interaction has not been consistent across studies [18,20,21]. Fasting glucose was not collected during the F1 exam cycle and we were unable to assess this interaction in our study.

This study is not without limitations. We are not able to evaluate clinical measures such as lipids, glucose, and insulin levels in this association, as they were not measured. These metabolic risk factors may be related to some of the pathways through which HbA1c may influence diabetes, and thus their adjustment may not be prudent. Related, adjustment for lipids has shown to have minimal effect on hazard ratios when collectively accounting for anthropometric and blood pressure measures [13,15,18,20,21]. The results from these previous studies suggest the potential confounding by unmeasured lipid values would not have a significant impact on the interpretation of our findings given the magnitude of our point estimates. Further, we found no meaningful change to our results after adjustment for regular diet and physical activity level, both traditional precedents to lipid levels and strong predictors of incident diabetes. We were only able to measure HbA1c once and while HbA1c reflects glycemia of the prior 2–3 months and is often used in the clinical setting, how representative this one-time measure is of an individual over extended time periods is less clear. HbA1c test results are impacted by the presence of blood disorders that shorten red blood cell lifespan or create abnormal structural changes to hemoglobin, may misclassify gestational diabetes cases compared to oral glucose tolerance testing (OGTT), and differ by race regardless of diabetes status; in the United States, whites are found to have the lowest mean HbA1c compared to African Americans or Hispanics [22–25]. Thus, assay results require careful interpretation for particular populations. Lifestyle factors of smoking status and alcohol use were self-reported, leading to residual confounding and misclassification, though in a prospective study such as this the misclassification is most likely non-differential. The reference group was chosen in part due to the limited number of diabetes cases that developed in individuals with HbA1c ≤ 5.7%. We were unable to clearly evaluate the association between HbA1c and incident disease at HbA1c levels below HbA1c 5.7%. Diabetes ascertainment in this population was by self-report of physician diagnosis, thus individuals with HbA1c values deemed diabetic by recent ADA standards were considered to be disease free at baseline. The highest category of percentage HbA1c represent individuals who would be considered diabetic at the start of follow-up by ADA standards. In addition to elevated HbA1c levels, these individuals were of older age and greater BMI at the start of follow-up and the relatively low percentage of individuals in this HbA1c category who go on to self-report diabetes status at F2 is of concern. Potentially this low reporting of disease may be a result of a myriad of factors including differential utilization of preventive health services by age, physician’s use of non-ADA diagnostic standards (i.e., excluding the use of OGTT and HbA1c values from diagnostic decision making), or participant misreport of diabetes status [26,27]. We aimed to address the claim that individuals potentially with underlying diabetes would be identified in the first three years post blood-draw, leading to spurious results. The HRs in the lower HbA1c groups remained relatively stable with the exclusion of the first three years of observation time. The corollary observed in the highest HbA1c group with this follow-up time exclusion, while overtly noticeable, does not influence the interpretation of the original results. Future studies on similar populations should continue to hone the appropriate HbA1c cut-point for diagnosis of type 2 diabetes and reasons why diabetes may be going undiagnosed.

The strengths of this study should be considered when interpreting our findings. This analysis was able to evaluate a spectrum of HbA1c values below the ADA threshold for diagnosing diabetes. This is a large Chinese population in which participant information was collected through detailed in-person interviews, with high participant response rates, diabetes case validation, and adjustment for potential confounders. The population of Singapore has experienced rapid industrialization and accelerated advances in medical care over the last 50 years since its independence, resulting in a relatively high standard of living. This offers a unique opportunity to study a population on the leading edge of westernization of diet and lifestyle still within Asia. The participants in this study are representative of the source population supporting the results observed being applicable to individuals of Hokkien and Cantonese descent living in this part of Southeast Asia.

In this population-based study of nondiabetic Singapore Chinese adult men and women, glycated hemoglobin level, below the diagnostic threshold for diabetes (HbA1c 5.8%-6.4%), independently predicts incident diabetes after adjustment for potential confounding factors. This study provides support for a continuum of risk for developing diabetes at HbA1c % levels below 6.5%. Therefore, the results of this study support the clinically established HbA1c values for individuals at increased risk for diabetes in Western populations.
